# Bioinformatics-Led Discovery of Osteoarthritis Biomarkers and Inflammatory Infiltrates

**DOI:** 10.3389/fimmu.2022.871008

**Published:** 2022-06-06

**Authors:** Xinyue Hu, Songjia Ni, Kai Zhao, Jing Qian, Yang Duan

**Affiliations:** ^1^Department of Clinical Laboratory, Kunming First People’s Hospital, Kunming Medical University, Kunming, China; ^2^Department of Orthopedic Trauma, Zhujiang Hospital, Southern Medical University, Guangzhou, China; ^3^Neurosurgery Department, The Second Affiliated Hospital of Kunming Medical University, Kunming, China; ^4^Department of Spine Surgery, Zhujiang Hospital, Southern Medical University, Guangzhou, China

**Keywords:** osteoarthritis, immune infiltration, biomarker, GEO, differentially expressed genes

## Abstract

The molecular mechanisms of osteoarthritis, the most common chronic disease, remain unexplained. This study aimed to use bioinformatic methods to identify the key biomarkers and immune infiltration in osteoarthritis. Gene expression profiles (GSE55235, GSE55457, GSE77298, and GSE82107) were selected from the Gene Expression Omnibus database. A protein-protein interaction network was created, and functional enrichment analysis and genomic enrichment analysis were performed using the Gene Ontology (GO) and Kyoto Encyclopedia of Genes and Genome (KEGG) databases. Immune cell infiltration between osteoarthritic tissues and control tissues was analyzed using the CIBERSORT method. Identify immune patterns using the ConsensusClusterPlus package in R software using a consistent clustering approach. Molecular biological investigations were performed to discover the important genes in cartilage cells. A total of 105 differentially expressed genes were identified. Differentially expressed genes were enriched in immunological response, chemokine-mediated signaling pathway, and inflammatory response revealed by the analysis of GO and KEGG databases. Two distinct immune patterns (ClusterA and ClusterB) were identified using the ConsensusClusterPlus. Cluster A patients had significantly lower resting dendritic cells, M2 macrophages, resting mast cells, activated natural killer cells and regulatory T cells than Cluster B patients. The expression levels of TCA1, TLR7, MMP9, CXCL10, CXCL13, HLA-DRA, and ADIPOQSPP1 were significantly higher in the IL-1β-induced group than in the osteoarthritis group in an *in vitro* qPCR experiment. Explaining the differences in immune infiltration between osteoarthritic tissues and normal tissues will contribute to the understanding of the development of osteoarthritis.

## Introduction

Osteoarthritis is a degenerative joint disease that affects the elderly the most (1). It is the major cause of disability and is characterized by cartilage destruction and bone fragmentation in joints (2, 3). Prior joint injury, abnormal joint or limb development, genetic background, and having a job that requires heavy lifting are among the risk factors for osteoarthritis caused by mechanical stress on the joints and low levels of inflammation (4). osteoarthritis is the most common chronic joint disease, which cannot be prevented, and its prevalence rises with age (5–7). The treatment costs for osteoarthritis have created a financial burden for patients and society, causing a serious threat to human health. The disease’s etiology and pathogenesis, however, are still unknown. To develop effective treatment strategies, it is necessary to investigate the underlying mechanisms of osteoarthritis development.

Animal models (7), tissue models (8), gene expression (9), and systems biology approach (10) have been used to investigate the molecular mechanisms of osteoarthritis. Many researchers have focused on the identification of disease-associated proteins for osteoarthritis, such as type 2 collagen(COL2A1) and aggregated proteoglycans(ACAN) (11), nucleotide-binding and oligomerized structural domain receptor inflammatory vesicles containing protein 3 (NLRP3) (12), and matrix metalloproteinase (MMP)-13 (13). The molecular basis of Osteoarthritis pathology, however, remains unexplained.

The involvement of differentially expressed genes (DEGs) and biofunctional pathways, which are analyzed using microarray and bioinformatic technologies, in the development of osteoarthritis microarray and bioinformatics has extended the scope of the previous genome-based studies to screening for genetic alterations (10) Bioinformatics is an interdisciplinary method used to study the molecular mechanisms of diseases (14). Improved knowledge of the molecular networks and genes involved in those networks will enhance the overall understanding of the pathology of osteoarthritis and the molecular networks and genes involved. High false positive rates were observed in the study that used only one microarray platform and a small number of samples, which may have contributed to the inconsistent results (15). Therefore, further research is required to identify new therapeutic targets and diagnostic biomarkers that are more reliable to overcome the inconsistencies observed in previous studies.

In this study, gene expression profiles and microarray experiments were used to analyze the genes that were significantly different between patients with osteoarthritis and control samples. The identification of key biomarkers of unusually expressed genes and immune infiltration will improve our understanding of the molecular mechanisms of osteoarthritis at the systems biology level.

## Materials and Methods

### Microarray Data Source

The datasets GSE55235 (16), GSE55457 (16), GSE77298 (17), and GSE82107 (18) were downloaded from the Gene Expression Omnibus (GEO) database. Microarray data of GSE55235 contain 20 arthritis samples and 10 control samples, those of GSE55457 contain 23 arthritis samples and 10 control samples, those of GSE77298 contain 16 arthritis samples and 10 control samples, and those of GSE82107 contain 16 arthritis samples and 10 control samples. The GSE55235 and GSE55457 datasets were sequenced on GPL96, GSE77298 and GSE82107 on GPL570, and GSE77298 and GSE82107 on GPL570, all with the human body as the origin. (**Table 1**) To create the integrated GEO dataset, the four datasets mentioned above were de-batched using the R package sva (19) to contain 69 arthritis samples and 34 control samples.

**Table 1:** Descriptive statistics.

Data number	Platform information	Osteoarthritis group	Control group	Species
GSE55235	GPL96	20	10	Homo sapien
GSE55457	GPL96	23	10	Homo sapien
GSE77298	GPL570	16	7	Homo sapien
GSE82107	GPL570	10	7	Homo sapien

The ImmPort (20), GenegCards (21), and MSigDB (22) databases were used to obtain 1509, 16,664, and 21,341 immune-related genes, respectively. Furthermore, 1264 immune-related genes were derived from the intersection of the aforementioned three gene sets.

### Identification of Arthritis-Related Immune-Differentially Expressed Genes

Differential gene analysis was performed using the R package “limma” (23) to determine the differential genes between diseased and control samples in the integrated dataset to investigate the impact of gene expression levels of immune-related genes on arthritis. The thresholds for differential genes were set at the absolute value of log2 fold change |log2FC| >1 and adj. *P <*0.05, indicating DEGs with upregulated expression. Similarly, log2FC <-1and adj. *P <*0.05 indicates DEGs with downregulated expression. Volcano plots and heat maps were used to display the results of differential gene expression. The differentially expressed immune genes were identified by intersecting DEGs and immune genes.

### Forest Model and Nomogram Model Construction

The model was trained using the least absolute shrinkage and selection operator (LASSO) technique to predict the likelihood of arthritis. The candidate differentially expressed immune genes were included in the model and analyzed using the LASSO algorithm to obtain the characteristic genes associated with arthritis. The risk score formula (Eq. 1) was established using the forest model to predict the likelihood of arthritis.


(1) Risk Score=(exp−Gene1*coef−Gene1)+(exp−Gene2*coef−Gene2)+…+(exp−Gene*coef−Gene)

The prevalence of arthritis in patients was then predicted using columnar line graph models based on the chosen candidate Imm-DEGs. The expected values were compared to the standard value using calibration curves. To determine whether the model-based decisions were beneficial to patients, decision curve analysis was performed and clinical impact curves were drawn.

### Identification of Molecular Subtypes Based on Important Immunomodulators

Consistency clustering is a resampling-based approach for identifying each member and their subgroup number, as well as validating the cluster. To discover various immunological patterns based on significant Imm-DEGs, the ConsensusClusterPlus package (24) in R was employed.

### Determination of DEGs in Various Immunological Patterns

To investigate the effect of diverse immunological patterns on arthritis, differentially expressed genes were screened for significant differential genes using the R package limma on immune pattern subgroups in the integrated dataset. Differentially expressed genes with |log2FC| >1 and adj. *P <*0.05 have upregulated expression, while those with log_2_FC=-1 and adj. *P <*0.05 has downregulated expression. The results of the differential gene expression were obtained using volcano and heat maps.

### Assessment of Biological Variables Among Various Immunological Patterns

Gene Ontology (GO) analysis is a standard method to conduct large-scale functional enrichment studies, covering biological processes, molecular functions, and cellular components (24). Kyoto Encyclopedia of Genes and Genome (KEGG) is a widely used database for storing information on genomes, biological pathways, diseases, and pharmaceuticals (25). GO annotation analysis and KEGG pathway enrichment analysis of differentially expressed genes were performed using R’s clusterProfiler package (26), and a threshold value of <0.05 for false discovery rate was considered statistically significant.

To investigate the differences in biological processes between different subgroups, based on a dataset of gene expression profiles from arthritis patients, we performed gene set enrichment analysis (GSEA). GSEA is a computational method used to analyze whether a particular gene set is statistically significant and consistent with the differences between two biological states (22). GSEA is commonly used to estimate the changes in the pathways and biological activities in the samples of expression datasets.The gene sets “c2.cp.kegg.v7.4.symbols.gmt” and “c5.go.v7.2.symbols.gmt” were retrieved from the MSigDB database for use in the GSEA. *P*-value <0.05 was considered statistically significant.

### Protein-Protein Interaction Network

Protein-protein interaction (PPI) networks are created for individual proteins interacting with one other to participate in numerous activities of life processes, such as biological signaling, control of gene expression, energy and material metabolism, and cell cycle regulation. Systematic analysis of the interactions of a large number of proteins in biological systems is important to understand how proteins work in biological systems. Systematic analysis is also useful to understand the response mechanisms of biological signaling and energy-matter metabolism in specific physiological states (disease) and functional connections between proteins. The STRING database (27) is widely used for searching for known proteins and predicting relationships between proteins. Using the STRING database, we constructed a PPI network linked with differentially expressed genes, Imm-DEGs, and prospective differentially expressed immune genes. The PPI network model was displayed using Cytoscape (v3.7.2) (28), and the genes in the network were functionally annotated using closeGO (29).

### Identification and Correlation of Disease Immune Infiltrate Cells

The immune microenvironment generally consists of immune cells, inflammatory cells, fibroblasts, mesenchymal samples, different cytokines, and chemokines. Immune cell infiltration analysis has an important guiding role in the prediction of the disease course and response to therapy. The single sample gene set enrichment analysis (ssGSEA) algorithm, an extension of the GSEA approach, was employed to quantify the abundance of 28 immune cell types in individuals with different immunological patterns (30). The CIBERSORT algorithm, which can perform linear support vector regression to deconvolute gene expression profiles, was used to estimate the number of immune cells in samples using RNA-sequencing data (31). We calculated 22 immune cell types in patients with distinct immunological patterns in the dataset using the CIBERSORT algorithm (31) in R software and showed the immune cell composition of patients with varied immune patterns using box plots. Differences in immune cell proportions were evaluated using the Wilcoxon rank-sum test. P <0.05 was considered statistically significant.

### qRT-PCR Validation of the Hub Genes

Interleukin-1β (IL-1β) can induce cartilage degradation by promoting the expression of matrix metalloproteinases (MMPs) in chondrocytes (32) and is widely used in the inflammatory induction model of chondrocytes in osteoarthritis (33) . We used IL-1β to stimulate chondrocytes of normal and osteoarthritis person to simulate this microenvironment of inflammation and used qRT-PCR to verify the expression level of hub genes. The chondrocytes from normal people (CP-H107, Procell, wuhan, Hubei, China) and patients with osteoarthritis (402OAK-05a, Haoge Biotechnology Co., Ltd, Shanghai, China) and were cultured in DMEM/F12 medium (SH30126.01, HyClone Technologies, Logan, USA) containing 10% fetal bovine serum (FBS, 10099, Thermo Fisher Scientific, Massachusetts, USA) in 5% CO_2_ at 37°C and divided into NC (negative control) group and IL-1β group. Cells in the NC group were added to the normal medium while cells in the IL-1β group were treated with IL-1β (40 ng/mL) culture medium. The mRNA relative expression of TCA1, TLR7, MMP9, CXCL10, CXCL13, HLA-DRA, ADIPOQ, LEP, and SPP1 were detected after 48 h of incubation. The total RNA of osteoarthritis was extracted and synthesized into cDNA according to the manufacturer’s protocol (Promega Biotech Co., Ltd, Beijing, China). qRT-PCR was performed on a LightCycler 96 (Roche Life Sciences, Switzerland, Basel) using Real-Time PCR Mix (Vazyme Biotech, Nanjing, Jiangsu Province, China). Gene expression relative to GAPDH expression was assessed using the 2^-ΔΔCt^ method. Independent experiments were conducted in triplicate. (The sequence fragments of RNAs are shown in **Table 2**).

**Table 2:** PCR primers.

Gene	Forward primer sequence	Reverse primer sequence
TAC1	ACTGGTCCGACTGGTACGACAG	AGAACTGCTGAGGCTTGGGTCT
TLR7	TCCTTGGGGCTAGATGGTTTC	TCCACGATCACATGGTTCTTTG
MMP9	GGCACCACCACAACATCACCTA	CGGGCAAAGGCGTCGTCAAT
CXCL10	ACCTCCAGTCTCAGCACCATGA	TGCAGGTACAGCGTACAGTTCT
CXCL13	GCTTGAGGTGTAGATGTGTCC	CCCACGGGGCAAGATTTGAA
HLA-DRA	AGTCCCTGTGCTAGGATTTTTCA	ACATAAACTCGCCTGATTGGTC
ADIPOQ	ATGCTGTTGCTGGGAGCTGTTC	ATGCCCGCCATCCAACCTGT
LEP	TGCCTTCCAGAAACGTGATCC	CTCTGTGGAGTAGCCTGAAGC
SPP1	CTCCATTGACTCGAACGACTC	CAGGTCTGCGAAACTTCTTAGAT
GAPDH	AAGTATGACAACAGCCTCAAG	TCCACGATACCAAAGTTGTC

The experiment was divided into 2 groups: Interleukin-1β (IL-1β) group cells were added to a drug-containing medium of 40 ng/mL; osteoarthritis group cells were added to the normal medium. The mRNA relative expression of *TCA1, TLR7, MMP9, CXCL10, CXCL13, HLA-DRA, ADIPOQ, LEP*, and *SPP1* were detected after 48 h of incubation. The primers were designed using the DNAMAN software and synthesized by Sangon Biotech (Shanghai, China), with primer sequences shown in **Table 2**. The cellular RNA was extracted using TRIzol (Invitrogen #15596-026). cDNA was synthesized using PrimeScript™ RT reagent Kit with gDNA Eraser (Takara#RR047A) and SYBR Green qPCR Mix (Beyotime#D7260). The 7500 Real-Time Polymerase Chain Reaction (RT-PCR) System was used to complete amplification in 40 cycles. The PCR data were processed using GAPDH as an internal reference, and the relative expression in the samples was calculated using the AGCT method.

### Statistical Analysis

All data processing and analysis were performed in RStudio (version 4.1.1). To compare two groups of continuous variables, statistical significance of normally distributed variables was calculated using an independent Student’s *t*-test, and differences between non-normally distributed variables were calculated using the Mann-Whitney U-test (i.e., Wilcoxon rank-sum test). The chi-square test or Fisher’s exact test was carried out to analyze the statistical significance between two sets of categorical variables. Correlation coefficients between different genes were estimated *via* Pearson correlation analysis. All statistical P values were two-sided, and *p <*0.05 was considered statistically significant.

## Results

### Expression of Immune-Related Genes in Arthritis Patients

**Figure 1:**
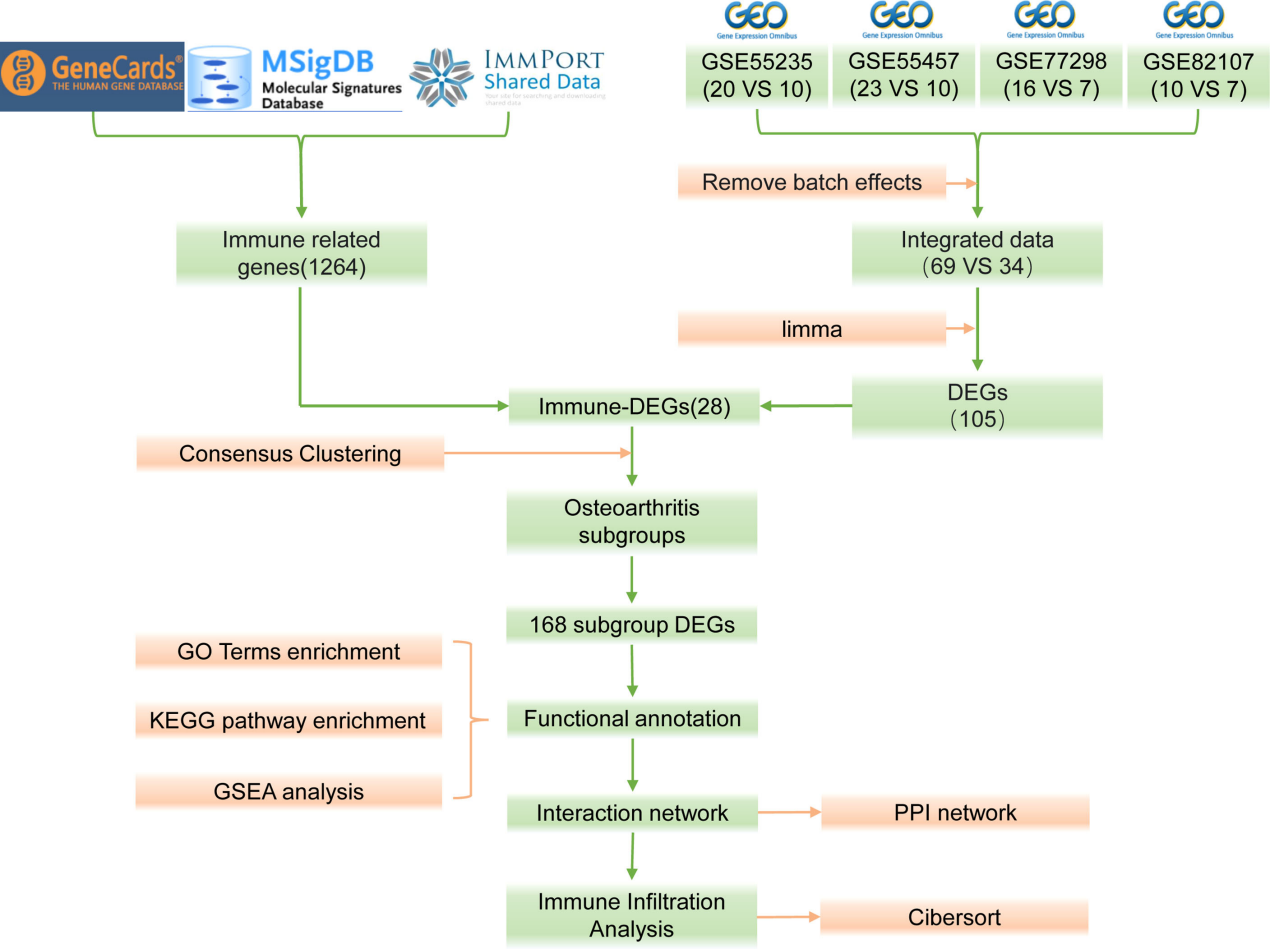
Flow chart.

**Figure 2:**
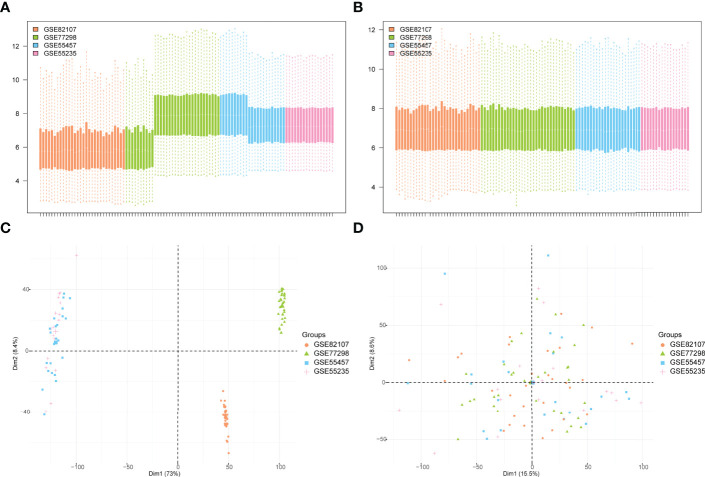
GEO data de-batching. **(A)** Gene expression level statistics of the dataset before de-batching. **(B)** Gene expression level statistics of the integrated dataset after de-batching. **(C)** Principal component analysis (PCA) between datasets before de-batching. **(D)** PCA between integrated datasets after de-batching.

**Figure 3:**
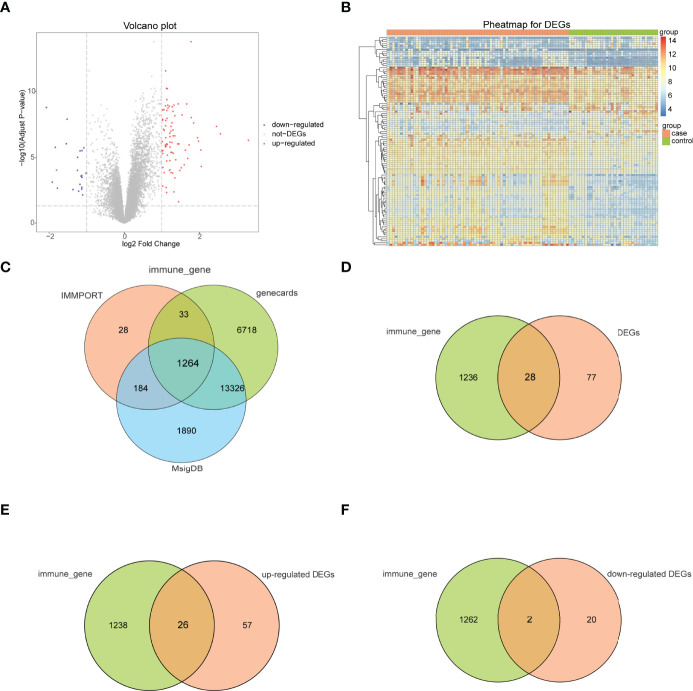
Immune-related genes and differentially expressed immune genes (Imm-DEGs). **(A)** Arthritis-related differentially expressed genes (DEGs) volcano plot with log2FoldChange in the horizontal coordinate and -log_10_(Adjust P-value) in the vertical coordinate. Red nodes indicate upregulated DEGs, blue nodes indicate downregulated DEGs, and gray nodes indicate genes that are not significantly differentially expressed. **(B)** Heat map of arthritis-related DEG expression levels: pink indicates disease samples, green indicates normal control samples, red indicates high gene expression, and blue indicates low gene expression. **(C)** Immune gene Venn diagram: three colors represent three different data sources. **(D)** Immune gene versus DEG Venn diagram: pink represents immune genes, green represents DEGs **(E)** Venn diagram of immune genes and upregulated DEGs: pink represents immune genes, green represents upregulated DEGs. **(F)** Venn diagram of immune genes and downregulated DEGs: pink represents immune genes, green represents downregulated DEGs.

The bioinformatics analysis of this study is carried out according to **Figure 1**. The batch effects were removed from the GEO dataset to obtain the integrated dataset (**Figure 2**), which includes 69 arthritis samples and 34 control samples. Differential study of arthritic samples and control samples revealed 105 DEGs; 83 of them were upregulated and 22 were downregulated (**Figures 3A, B**). **Figure 3C** shows the intersection of immune-related genes from three datasets and the intersection of DEGs on immune-related genes. These intersections yielded 28 Imm-DEGs (**Figure 3D**), 26 of which had an upregulated expression pattern (**Figure 3E**) and 2 had a downregulated one (**Figure 3F**).

**Figure 4:**
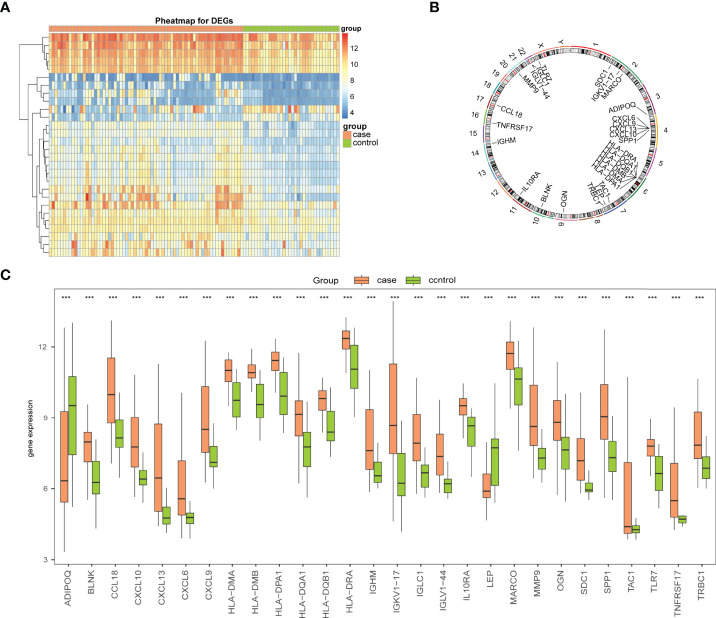
Expression levels of Imm-DEGs in arthritis. **(A)** Heat map of overall expression of Imm-DEGs in arthritis patients: green for control samples, pink for disease samples, red for high expression, blue for low expression. **(B)** Chromosome distribution of immune-related genes in arthritis patients. **(C)** Overall expression histogram of immune-related genes in arthritis patients: green for control samples, pink for disease samples, horizontal axis indicates genes, vertical axis indicates gene expression levels. (***P < 0.001).

To analyze the overall expression of Imm-DEGs, heat maps (**Figure 4A**) and histograms of the expression levels of Imm-DEGs in arthritis samples and control samples were drawn. Most genes were expressed at higher levels in arthritis samples than in normal samples (**Figure 4C**), and the positions of Imm-DEGs were annotated on human chromosomes (**Figure 4B**).

### Construction of Risk Model

**Figure 5:**
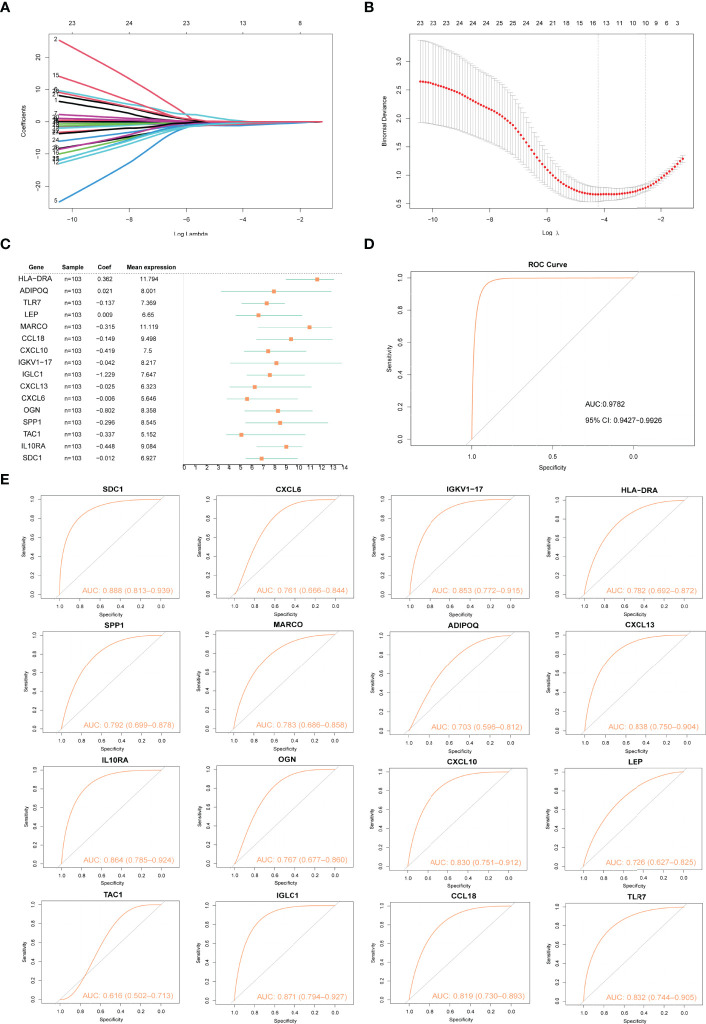
Construction of arthritis model. **(A, B)** Screening of gene signatures from Imm-DEGs using the LASSO algorithm. **(C)** Forest plot of gene signatures in arthritis patients. **(D)** Receiver operating characteristic (ROC) curve of predicted risk scores in arthritis diagnosis. **(E)** ROC curve of 16 gene signatures in arthritis diagnosis.

The LASSO algorithm was used to identify 16 characteristic genes out of 28 Imm-DEGs with a great effect on arthritis (**Figures 5A, B**). Based on the coefficients of 16 characteristic genes (**Figure 5C**), the gene expressions were multiplied by the corresponding coefficients and summed to derive the arthritogenic score (**Figure 5D**). Similarly, the receiver operating characteristic (ROC) curves of 16 gene signatures were investigated to predict arthritis, and the findings revealed the predictive efficacy of all 16 gene signatures (**Figure 5E**).

**Figure 6:**
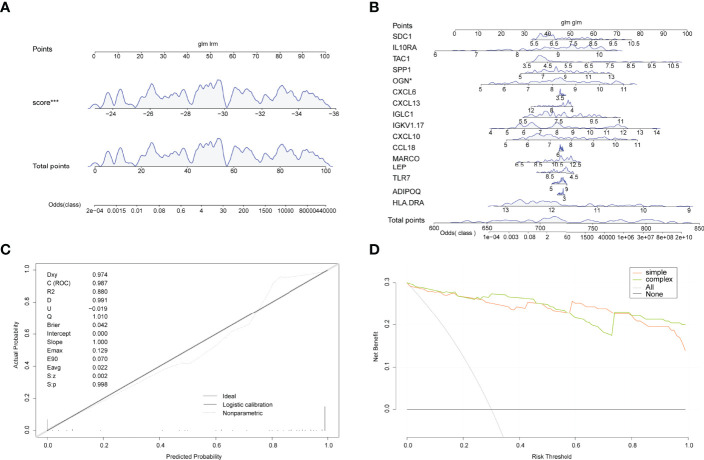
Line graphs (nomogram plots). **(A)** Nomogram of predicted risk scores in the diagnosis of arthritis patients. **(B)** Nomogram of 16 trait genes in the diagnosis of arthritis patients. **(C)** Nomo model evaluation, where the diagnostic model is in better agreement with the ideal model. **(D)** Model evaluation curves: gray indicates the follow-on diagnosis, green indicates the complex diagnostic model with 17 trait genes, and pink indicates the simple diagnostic model with predicted risk score. (*P<0.05, ***P<0.001).

A line plot model was created based on the patient’s projected risk score and 16 trait genes to predict the prevalence of arthritis in patients (**Figures 6A, B**). The calibration curves revealed that the line graph model predictions were nearly identical to those of the ideal model (**Figure 6C**) and that the single predicted risk score in the decision curve analysis or the composite genetic model is better than that in the random model. These results imply that decision-making based on the line graph model could be beneficial for arthritis patients (**Figure 6D**).

**Figure 7:**
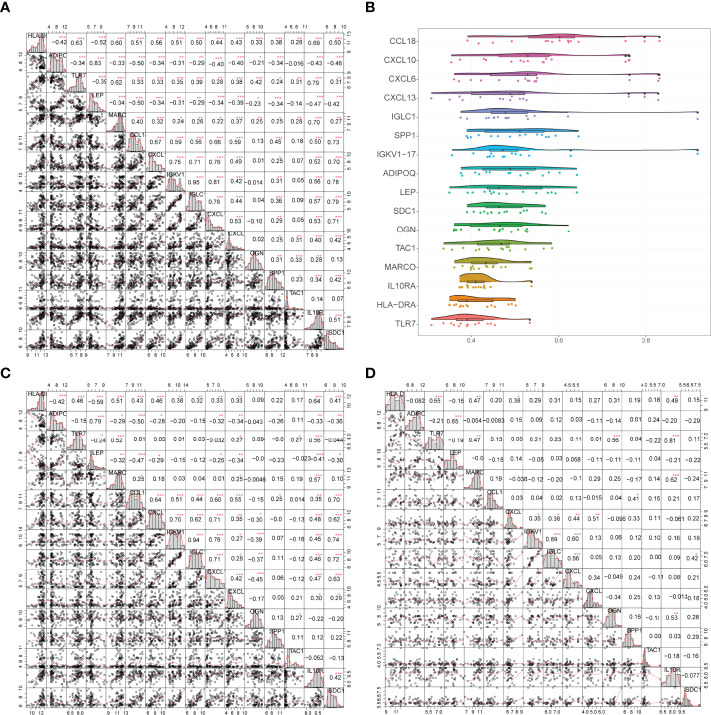
**(A)** Correlation analysis of 16 genes in all samples: * represents the significance of the correlation, and the number represents the correlation level. **(B)**: Functional correlation analysis of 16 trait genes, the horizontal axis indicates the correlation size and the vertical axis indicates the trait genes. **(C, D)** Correlation analysis of 16 trait genes in disease and normal samples: * represents the significance of correlation, and the number represents the correlation level. (*P<0.05, **P<0.01, ***P<0.001).

Correlations between gene expression levels and functional correlations between 16 gene signatures were examined. Coefficients of functional correlations between IGKV1-17, IGLC1, and other genes reached 0.9 (**Figure 7B**). In all samples, the correlation coefficient between IGKV1-17 and IGLC1 was 0.95; ADIPOQ had a positive correlation with LEP but a negative correlation with all other genes except ADIPOQ, and LEP had a negative correlation with all other genes except ADIPOQ (**Figure 7A**). In arthritic samples, the correlation coefficient between IGKV1-17 and IGLC1 was 0.94; ADIPOQ showed a positive correlation with LEP and SPP1 and a negative correlation with all other genes except ADIPOQ. Similarly, LEP showed a negative correlation with all other genes except ADIPOQ (**Figure 7C**). *OGN* gene also showed a negative correlation with most other characteristic genes. In control samples, the correlation coefficient between IGKV1-17 and IGLC1 was 0.89 (**Figure 7D**).

### Distinct Immunological Patterns of Gene Signatures

**Figure 8:**
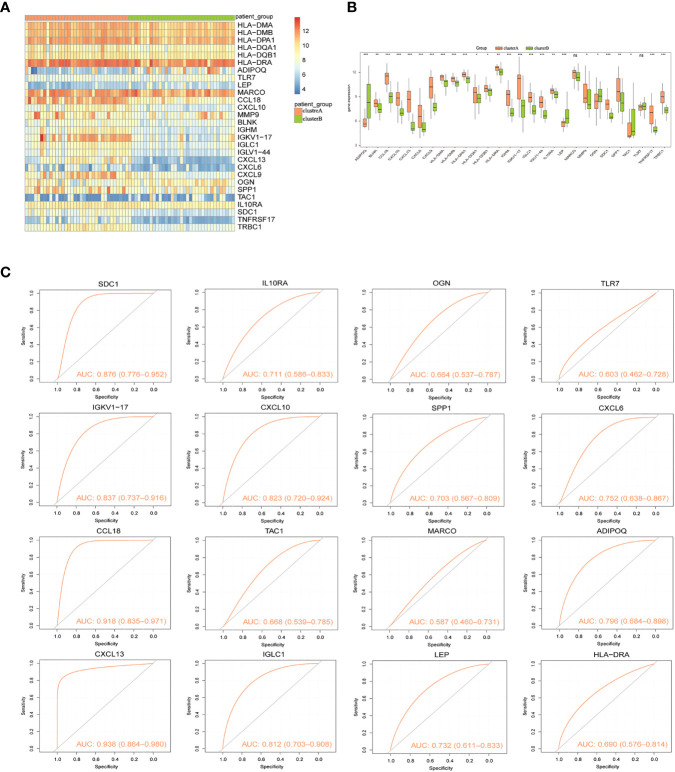
Consistency clustering of gene signatures in arthritis patients. **(A)** Heat map of expression levels of 28 Imm-DEGs in two clusters: pink indicates cluster A, green indicates cluster B, red indicates high expression, and blue indicates low expression. **(B)** Expression levels of 28 Imm-DEGs in two clusters: pink indicates cluster A, green indicates cluster B, the horizontal axis is the Imm-DEGs, and the vertical axis is the gene expression level. **(C)** ROC curves of 16 characterized genes independently distinguished between cluster A and cluster B. (nsP≥0.05, *P<0.05, **P<0.01, ***P<0.001).

Two immunological patterns (clusterA and clusterB) were established utilizing the ConsensusClusterPlus package in R software and a consistent clustering approach based on 16 signature genes. There were 34 samples in Cluster A and 35 samples in Cluster B. Heat maps for all differentially expressed immune genes were then created to show the considerable differences in immune gene expression between the two groupings (**Figure 8A**). Expression levels of *ADIPOQ, LEP, OGN*, and *TAC1* were significantly lower in cluster A than that in cluster B, whereas *HLA-DMA, HLA-DMB, HLA-DPA1, HLA-DQA1, HLA-DQB1 HLA-DRA, TLR7, CCL18, CXCL10, MMP9, BLNK, IGHM, IGKV1-17, IGLC1, IGLV1-44, CXCL13, CXCL6, CXCL9, SPP1, IL10RA, SDC1, TNFRSF17*, and *TRBC1* expression levels were significantly higher in cluster A than that in cluster B (**Figure 8B**). Similarly, the ROC curves of the 16 gene signatures individually predicted for both categories were evaluated, and the findings showed that all 16 gene signatures had good classification efficacy (**Figure 8C**).

### PPI Network of Immune Genes

**Figure 9:**
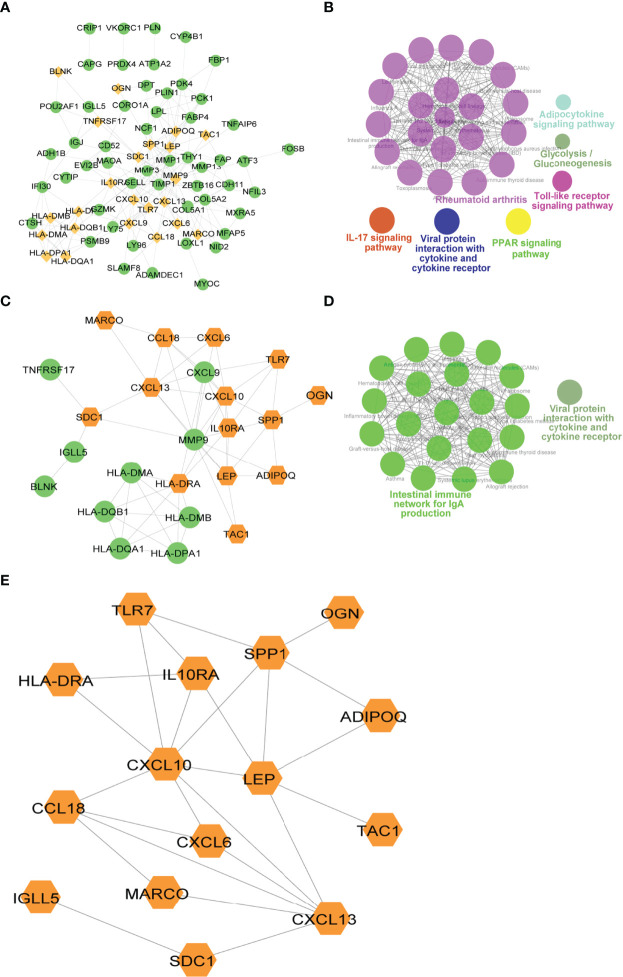
Immune-related gene protein-protein interaction (PPI) network. **(A)** Differentially expressed gene PPI network: yellow nodes indicate Imm-DEGs. **(B)** Results of gene enrichment analysis in DEG PPI network. **(C)** Differentially expressed immune gene PPI network: yellow nodes indicate gene signatures. **(D)** Results of gene enrichment analysis in Imm-DEG PPI network. **(E)** Results of gene signatures.

To explore the relationship between differentially expressed immune genes, we extracted the PPI network of DEGs, Imm-DEGs, and gene signatures. As visualized in Cytoscape, the PPI network of DEGs had 211 pairing interactions and 75 genes; *MMP9* was strongly correlated with 19 DEGs, whereas *CXCL10* was linked to 16 DEGs (**Figure 9A**). Similarly, the PPI network of Imm-DEGs comprised 58 reciprocal pairs and 24 genes; *CXCL10* and *MMP9* were closely linked to 10 differentially expressed immune genes, whereas *CXCL13* and *HLA-DRA* were both linked to 8 differentially expressed immune genes (**Figure 9C**). The PPI network of the gene signatures contained 25 interaction pairs and 15 genes, where *CXCL10* and *MMP9* were closely linked to 10 Imm-DEGs, whereas *CXCL13* and *HLA-DRA* were both linked to 8 Imm-DEGs (**Figure 9E**). To verify the functions of genes in three PPI networks, functional enrichment analysis was performed using ClueGO. The results revealed that genes in the PPI network were enriched in the pathways of adipocytokine signaling, glycolysis/gluconeogenesis, toll-like receptor signaling, viral protein interaction with cytokine and cytokine receptor IL-17 signaling, and rheumatoid arthritis (**Figure 9B**). Gene enrichment in the PPI network for differentially expressed immune genes was involved in the intestinal immune network for IgA production, viral protein interaction with cytokine and cytokine receptors (**Figure 9D**).

### Differential Analysis of Two Different Immune Patterns

**Figure 10:**
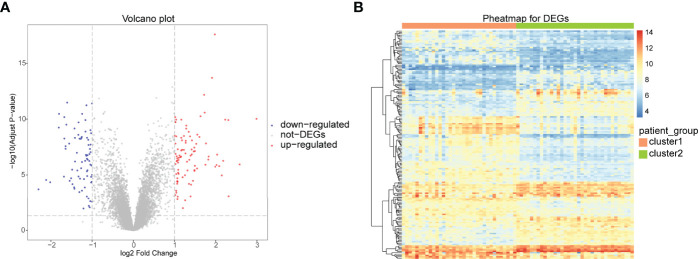
Differential analysis between two different immune patterns. **(A)** Horizontal coordinate is log2FoldChange; vertical coordinate is -log_10_ (Adjust P-value); red nodes indicate upregulated DEGs; blue nodes indicate downregulated DEGs; gray nodes indicate genes that are not significantly differentially expressed. **(B)** Heat map of expression levels of DEGs in two clusters: pink indicates cluster A; blue indicates cluster B; red indicates high expression; blue indicates low expression.

To analyze the differences between the two immune patterns, 168 DEGs were obtained between the patterns: cluster A and cluster B, including 93 DEGs upregulated and 75 DEGs downregulated in cluster A (**Figure 10A**). The heat maps showed that these DEGs could distinguish between the two different immune patterns (**Figure 10B**).

**Figure 11:**
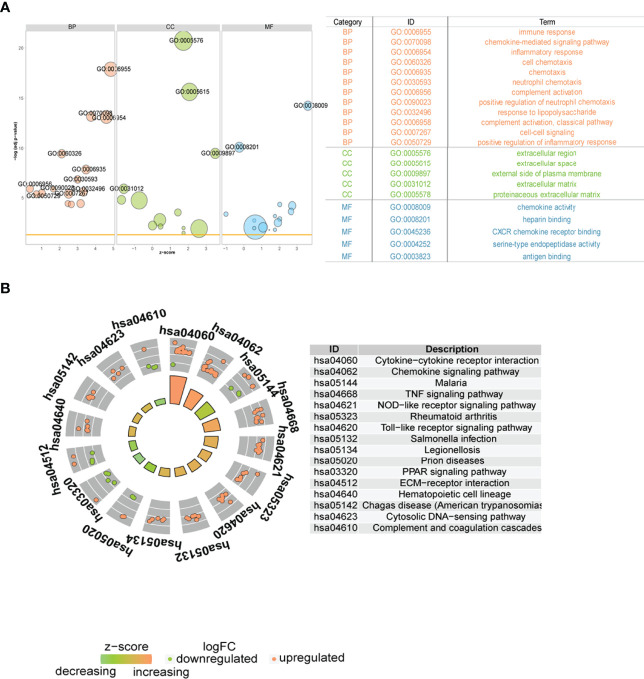
Functional analysis between two different immune patterns. **(A)** Gene Ontology (GO) functional enrichment analysis: vertical coordinate is the significance of the enrichment result; horizontal coordinate is the Z-score; node color indicates BP, CC, MF; node size indicates the number of genes contained in the current GO term. **(B)** Kyoto Encyclopedia of Genes and Genomes pathway enrichment analysis results: node color indicates gene expression level; quadrilateral color indicates Z-score. BP, Biological Process; MF, molecular function; CC, cellular component.

Subsequently, we analyzed the role of DEGs between the two immune modalities in the biologically relevant functions of patients. First, the DEGs were functionally annotated (**Figure 11A**), Second, these DEGs were enriched in cytokine-cytokine receptor interaction, chemokine signaling, malaria, and tumor necrosis factor (TNF) signaling, and NOD-like receptor signaling pathways upon KEGG pathway analysis (**Figure 11B**).

**Figure 12:**
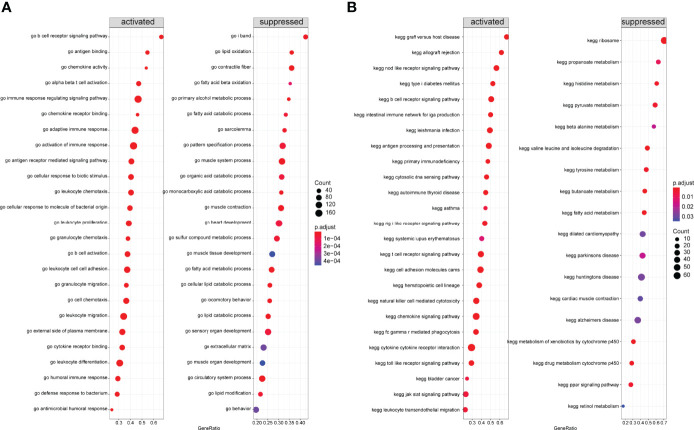
Gene Set Enrichment Analysis **(**GSEA) between two different immune models. **(A)** GSEA-GO analysis: horizontal coordinate is the gene ratio; vertical coordinate shows the GO terms; color indicates -log_10_ (p-value); node size indicates the number of genes enriched in GO terms. **(B)** GSEA-KEGG analysis: horizontal coordinate is the gene ratio; vertical coordinate shows the GO terms; node size indicates the number of genes enriched in the pathway; node color indicates -log_10_ (p-value).

Next, we performed GSEA on all genes between two immune modalities, which were significantly different in biological processes such as activation of immune response in cluster A, adaptive immune response based on somatic recombination of immune receptors built from immunoglobulin superfamily domains, alpha-beta T cell activation, and antigen binding. On the contrary, biological processes such as primary alcohol metabolic processes, regulation of insulin-like growth factor receptor signaling pathway, lipid oxidation, and retinol metabolism were inhibited (**Figure 12A**). Chemokine signaling pathway, cytokine receptor interaction, graft versus host disease, leishmanial infection, and NK cell-mediated cytotoxicity were activated, whereas butanoate metabolism, metabolism of xenobiotics by cytochrome p450, fatty acid metabolism, valine leucine and isoleucine degradation, and drug metabolism cytochrome p450 pathways were inhibited (**Figure 12B**).

### Differences in Immune Characteristics Between Two Models

**Figure 13:**
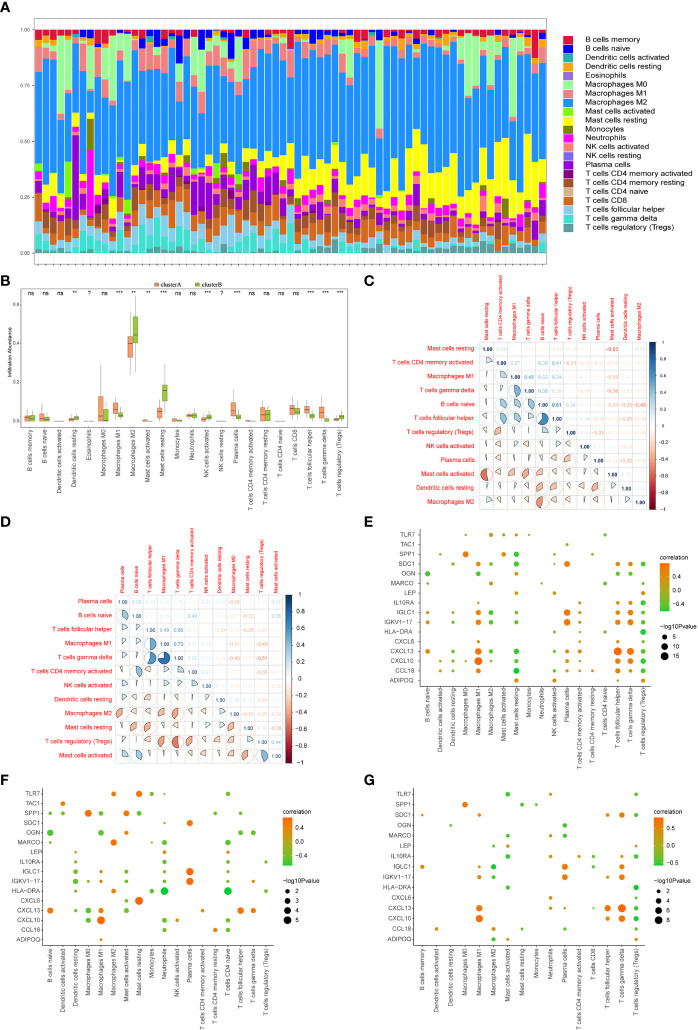
Immune characteristics between two different immune patterns. **(A)** Immune cell content stacking plot between cluster A and cluster B: different colors indicate different immune cells; the horizontal axis is the patient ID. **(B)** Immune cell content histogram: the horizontal axis indicates 22 immune cells; the vertical axis indicates cell content; pink indicates cluster A samples; green indicates cluster B samples. **(C)** Immune cell content histogram: the horizontal axis indicates 22 immune cells; the vertical axis indicates the cell content; pink indicates cluster A samples; green indicates cluster B samples. Correlations between 12 immune cells that significantly differed between cluster A and cluster B patients in the limma algorithm: correlations in cluster A patient species **(C)**; correlations in cluster B patient species **(D)**; red indicates negative correlations; blue indicates positive correlations. Correlations in all arthritis patients. Correlation analysis of 16 gene signatures and immune cell content in all arthritis patients **(E)**, cluster A arthritis patients **(F)** and cluster B arthritis patients **(G)**, horizontal axis indicates immune cells, vertical axis indicates 16 gene signatures, node color indicates the correlation size, and node size indicates the significance level. (?:no data, nsP≥0.05, **P<0.01, ***P<0.001).

The CIBERSORT algorithm was used to assess the level of immune cell infiltration between two different immune modalities. CIBERSORT analysis revealed that patients in cluster A had significantly lower levels of resting dendritic cells, M2 macrophages, resting mast cells, activated NK cells, and regulatory T cells than those in cluster B (**Figure 13A**). However, the levels of M1 macrophages, activated mast cells, plasma cells, T follicular helper cells, and gamma delta T cells were significantly higher than those in cluster B (**Figure 13B**).

The correlation between the immune cell contents of patients in clusters A and B was also calculated. The patients in cluster A showed a higher proportion of T follicular helper cells than that naïve B cells. Gamma delta T cells were in higher proportion than M1 macrophages. Furthermore, T follicular helper cells and activated memory CD4 T cells showed a significant positive correlation, whereas activated and resting mast cells, M2 macrophages, and naive B cells showed a significant negative correlation (p < 0.05, **Figure 13C**). Similarly, in cluster B patients, gamma delta T cells, M1 macrophages, T follicular helper cells, gamma delta T cells, and activated mast cells showed a positive correlation with regulatory T cells, whereas regulatory T cells showed a negative correlation with M1 macrophages, regulatory T cells, and gamma delta T cells (*p <*0.05, **Figure 13D**).

The correlations between 16 gene signatures and immune cell types were also calculated for all arthritis patients (**Figure 13E**), cluster A (**Figure 13F**), and cluster B arthritis patients (**Figure 13G**). TAC1 was not correlated with the immune cell content in cluster B arthritis patients, and resting memory CD4 T cells and activated NK cells were not correlated with the gene signatures (**Figure 13G**).

### qPCR Validation of Data

**Figure 14:**
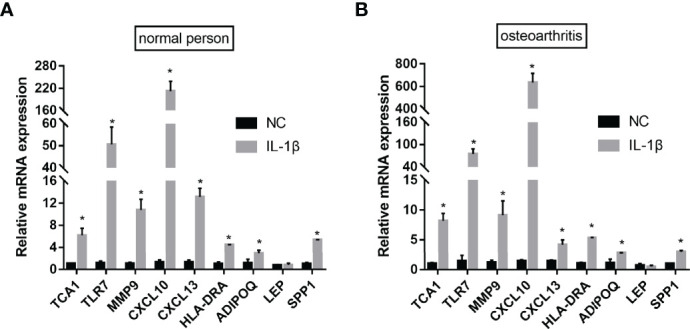
qPCR validation. After IL-1β-induced, the mRNA expression levels of TCA1, TLR7, MMP9, CXCL10, CXCL13, HLA-DRA, ADIPOQ and SPP1 were significantly higher both in the normal person** (A)** and osteoarthritis **(B)**. (nsP≥0.05, *P<0.05).

To verify the bioinformatics results, qPCR experiments were conducted. The results revealed that the mRNA expression levels of TCA1, TLR7, MMP9, CXCL10, CXCL13, HLA-DRA, and ADIPOQSPP1 were significantly higher in the IL-1β-induced group, with the most significant difference in the CXCL10 expression. The difference in the LEP expression between the two groups was not statistically significant. This indicates that the results of data mining are reliable and have potential research value (**Figure 14**).

## Discussion

Microarray technology and high-throughput technology, the main approaches to exploring the expression levels of genes, have improved the understanding of the intrinsic molecular mechanisms associated with complex disorders. Osteoarthritis is a degenerative joint condition that commonly affects the hands and weight-bearing joints (34). It is the most common joint disease in the world (35), affecting approximately 10–15% of the youth and 60% of the elderly (36). The molecular mechanism of its immune milieu is poorly understood; therefore, finding a novel target for early diagnosis, treatment, and prognosis of the immune microenvironment of osteoarthritis has important clinical benefits. All arthritis and control samples in the present study comprised 105 differently expressed genes; 28 of them were immune-related genes that overlapped with differentially expressed genes. Inflammatory response, immunological response, and the chemokine-mediated signaling pathway were the most highly enriched categories in the GO analysis of DEGs. Differentially expressed genes were found to be involved in cytokine-cytokine receptor interactions, chemokine signaling pathways, and malaria, as revealed by the KEGG analysis. Cluster A patients had significantly lower levels of resting dendritic cells, M2 macrophages, resting mast cells, and activated NK cells than cluster B patients did, whereas M1 macrophages, activated mast cells, plasma cells, and T follicular helper cells in cluster A were significantly higher than those in cluster B patients.

In the PPI reciprocal network analysis, *CXCL13, CXCL9, SPP1*, and *CXCL10* were linked to Imm-DEGs. Osteoarthritic osteoblasts directly regulate proliferation and type I collagen expression by CXCL13 chemokines (37). As reported in rheumatoid arthritis, proinflammatory cytokines enhance secondary CXCL13 production by reactivating CXCL13-producing CD4 T cells (38). Similarly, serum CXCL13 correlates with synovial CXCL13 measured at the joints, suggesting that synovitis is an important source of circulating CXCL13 (39). Past evidence showed the linkage between tissue bone bridging protein (SPP1) and osteoarthritis (40). Osteoarthritis-induced chondrocyte apoptosis is prevented by microRNA-186 inhibition of the PI3K-AKT pathway *via* SPP1 (41). *CXCL9* gene and protein expression were higher in proportion in the synovium of rheumatoid arthritis patients than that in osteoarthritis patients in a previous study (42). The same conclusions were drawn in a related study on the microarray analysis of gene expression in rheumatoid arthritis joints performed using cDNA microarrays and laser microdissection: CXCL9 and CXCL10 upregulation in the synovial lining associated with inflammation (43).

In GO and KEGG analyses, DEGs were enriched in immune response, chemokine-mediated signaling pathway, inflammatory response, chemokine activity, heparin-binding, antigen binding, cytokine-cytokine receptor interaction, chemokine signaling pathway, TNF signaling pathway, and NOD-like receptor signaling pathway. For receptor-chemokine interactions, the classical “two-site model” suggests that the globular core of chemokines serves as a docking structural domain of the receptor N-terminus (CRS1) that provides binding affinity, and chemokine N-terminus serves as a signaling trigger for the splice receptor binding pocket (CRS2) (44). One of the most important players in the disease process of two inflammatory joint diseases, rheumatoid arthritis and osteoarthritis, is the chemokine and chemokine receptor system (45). A recent study observed an increased expression of CC chemokine ligand 20 (a chemokine capable of binding to the CC chemokine receptor 6 expressed on Th17 cells) in inflamed joints of osteoarthritis patients (44). Cytokine-cytokine receptor interactions and rheumatoid arthritis are both triggered during the post-injury period (46). In addition, rheumatoid arthritis and osteoarthritis are caused by pro-inflammatory cytokines, which are released by the immune system in response to injury or inflammation (47). A local inflammatory process has been observed in osteoarthritic joints, and it is hypothesized that traumatic stimuli cause chondrocytes to produce cytokines and matrix metalloproteinases, which in turn lead to cartilage degradation in joints (48). A previous study confirmed the implication of inflammatory cytokines, TNF and IL-1, in the progression of rheumatoid arthritis and osteoarthritis (49).

Osteoarthritis is primarily a degenerative disease, rheumatoid arthritis is an autoimmune disease driven primarily by a significant inflammatory response involving the innate and adaptive immune systems (50). Inflammatory pathways of the innate immune system are activated in osteoarthritis because of the cellular stress and extracellular matrix degradation triggered by minor and major injury (51). A mosaic-like pattern of cytokine signaling and activation of molecular inflammatory pathways in the natural cells of intra-articular tissue pathogenesis contributes to the pathogenesis of osteoarthritis (52). Hence, The treatment of osteoarthritis through the modulation of the immune response is critical (53). In line with these findings, the GSEA analysis in this study revealed that osteoarthritis is predominantly linked to immune response activation. Furthermore, the GSEA data revealed a strong link to fatty acid metabolism. An important component of pathogenesis of osteoarthritis is the presence of intra-articular adipose tissue (54). Osteoarthritis is characterized by mitochondrial dysfunction and oxidative stress damage (55). For this reason, osteoblasts in human bone flap specimens from patients with osteoarthritis were studied and found to contain both *in-situ* active fatty acid oxidation and tricarboxylic acid metabolism (56). In osteoarthritis and other inflammatory disorders, fat pads contribute to the generation of adipokines and cytokines. This study and a previous study (57) demonstrated that adipokines regulate osteoarthritis and bone remodeling. This study’s GSEA analysis revealed that osteoarthritis was linked to graft-versus-host disease and Leishmania protozoa infection, which was one of the study’s findings.

The gene expression profile was subjected to Consensus Clustering by ConsensusClusterPlus package. In the omics analysis of large samples, it is often necessary to discuss the molecular typing of samples. The most common method in this paper is to cluster transcriptome, proteome and other data by Consensus Clustering. Finally, the samples can be divided into different clusters. There are obvious differences in transcriptome, proteome and other molecular patterns among the samples in different clusters, but the molecular patterns of the samples in each cluster are similar, thus realizing the purpose of molecular typing of large sample queues (58). For example, in the document “Proteogenomic landscape of squamous cell lung cancer”, based on the quantitative proteomic data of 108 lung squamous cell carcinoma samples, the author divided 108 tumor tissues into five molecular subtypes by consistent clustering, namely inflammatory subtype A, inflammatory subtype B, redox subtype A, redox subtype B and the molecular subtypes are obtained, the detailed subtype characteristics are described and discussed (59). A total of 494 lung squamous cell carcinoma samples were obtained from The Cancer Genome Atlas (TCGA) database. According to the proportion of immune cells identified in the samples, the tumor samples were analyzed by consistent clustering, and three subtypes were obtained. The subtypes I, II and III contained 17, 25 and 24 tumor samples, respectively. The clinical survival prognosis of subtype II is poor, while that of subtype I and III is good (60). In this study, we use consensus clustering to identify two immune patterns (clusterA and clusterB). ClusterA contains 34 samples and clusterB contains 35 samples. Through consistent cluster analysis and immune analysis, it is confirmed that there is significant heterogeneity between the two subgroups and the differential expression of related genes in the two subgroups. In the future, we will further study the specific mechanism of these genes in the immune microenvironment of osteoarthritis.

This study has some limitations. Firstly, we only studied the biological function of osteoarthritis in cells cultured *in vitro*, and further investigation of the unique processes is required. Second, although the aim of our study was to provide a landscape of mRNA and protein levels in osteoarthritis patients, we did not include more samples in our analysis (e.g., other rheumatic diseases). Future research should include a large number of samples and single cells. Additional histology (lipidomics, metabolomics, glycomics) and laboratory experiments may also improve the understanding of the conditions.

In summary, bioinformatics studies were performed to compare immune infiltration in the osteoarthritis and control groups. TCA1, TLR7, MMP9, CXCL10, CXCL13, HLA-DRA, and ADIPOQSPP1 were expressed at significantly higher levels in the IL-1β-induced group than in the osteoarthritis group, and our data also suggest that osteoarthritis may be associated with immune responses, chemokine-mediated signaling pathways, and inflammatory responses, and these studies improve our understanding of the development of osteoarthritis. The results of this study will aid in explaining the immune regulatory network of arthritis and inspire more effective therapeutic approaches. However, the functions of and associations between important genes and immune infiltration, as well as the significance of immune infiltration patterns in the development of osteoarthritis require further investigation.

## Data Availability Statement

The datasets presented in this study can be found in online repositories. The names of the repository/repositories and accession number(s) can be found in the article/supplementary material.

## Author Contributions

Data curation: KZ. Formal analysis: SN, YD. Methodology: SN, JQ, YD. Writing – original draft: XH. Writing – review and editing: XH, YD. All authors contributed to the article and approved the submitted version.

## Conflict of Interest

The authors declare that the research was conducted in the absence of any commercial or financial relationships that could be construed as a potential conflict of interest.

## Publisher’s Note

All claims expressed in this article are solely those of the authors and do not necessarily represent those of their affiliated organizations, or those of the publisher, the editors and the reviewers. Any product that may be evaluated in this article, or claim that may be made by its manufacturer, is not guaranteed or endorsed by the publisher.

## Abbreviations

BP, Biological Process; MF, molecular function; CC, molecular function; GEO, Gene Expression Omnibus; GO, Gene Ontology; KEGG, Kyoto Encyclopedia of Genes and Genomes.
